# Massive Secondary Postpartum Hemorrhage with Uterine Artery Pseudoaneurysm after Cesarean Section

**DOI:** 10.1155/2013/285846

**Published:** 2013-04-04

**Authors:** Ahmet Ozgur Yeniel, Ahmet Mete Ergenoglu, Ali Akdemir, Elmin Eminov, Fuat Akercan, Nedim Karadadaş

**Affiliations:** Department of Obstetrics and Gynecology, Ege University, Faculty of Medicine, Bornova, 35100 İzmir, Turkey

## Abstract

Uterine artery pseudoaneurysm is a rare but serious complication of cesarean section. If inadequately treated, it can lead to life-threatening postpartum hemorrhage. Herein, we report the case of a 28-year-old woman who developed secondary postpartum hemorrhage resulting from uterine artery pseudoaneurysm and cesarean scar dehiscence after cesarean section. Angiographic embolization is a safe and effective procedure for treating postpartum hemorrhage resulting from pseudoaneurysm in hemodynamically stable patients. However, uterine artery ligation may be the surgical procedure of choice for hemodynamically unstable patients when fertility preservation is desired.

## 1. Introduction

Uterine artery pseudoaneurysm (UAP) is a rare but life-threatening complication of uterine surgery, especially in cesarean section (C/S) [1, 2]. This condition may result in secondary postpartum hemorrhage, which is defined as hemorrhage that occurs between 24 hours and 6–12 weeks postpartum [3]. Although a diagnosis of retained gestational products or endometritis should be considered initially, a diagnosis of UAP and cesarean scar dehiscence (CSD) should be considered when a patient presents with massive uterine bleeding without any associated symptoms such as fever and tenderness or subinvolution of the uterus. Hematoma formation involving the uterine artery is the main suggested mechanism associated with UAP. UAP can be differentiated from true aneurysm by performing a histopathological examination. Turbulent blood flow on color Doppler sonography may be the single diagnostic finding in asymptomatic patients, and the absence of a 3-layer arterial wall lining is the most important histopathological finding that distinguishes UAP from true aneurysm [4, 5]. Proper treatment requires an accurate diagnosis, which is generally based on the results of color Doppler sonography and confirmed by performing angiography. Arterial embolization should be considered as the treatment of choice for stable patients [6]. 

Herein, we report a case representing coexistence of UAP and CSD with the presence of massive uterine bleeding managed by a fertility preserving surgical approach. To our knowledge, this is the first reported case “coexistence of UAP and CSD as the cause of delayed postpartum hemorrhage” which was treated by surgical procedure. 

## 2. Case Report

A 28-year-old patient (gravida 2 para 2) who delivered a 3580 g male fetus by cesarean section a month ago was referred to our clinic with postpartum hemorrhage. The course of 2 previous C/S was unremarkable. She was discharged from the hospital on the second day postpartum, and on the third week postpartum, she was admitted to the hospital with complaint of severe vaginal bleeding.

**Figure 1:**
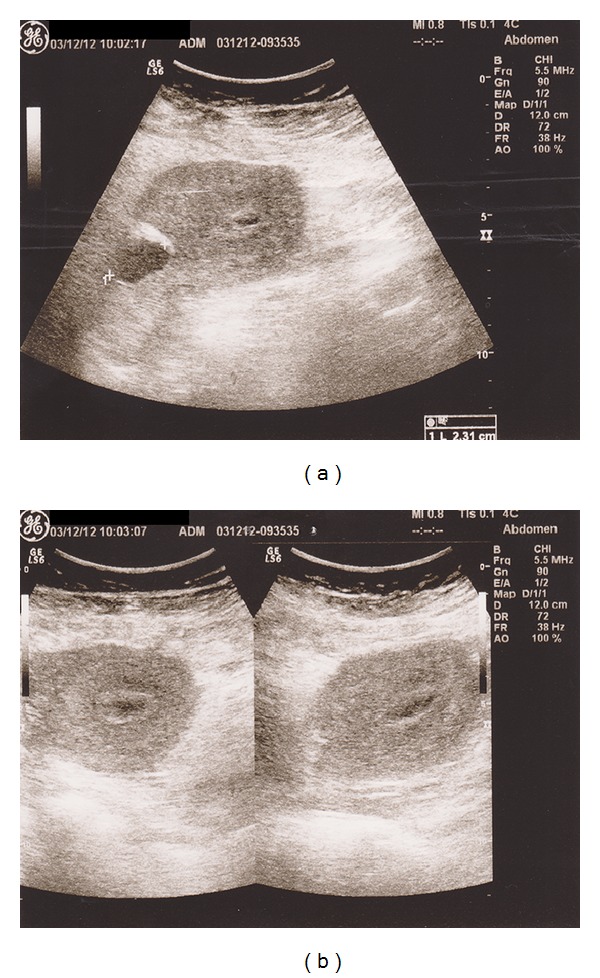
A lesion, 20 mm in diameter, consistent with fluid collection, was detected on gray scale sonography.

Initial evaluation of the patient revealed tachycardia (120 bpm) and paleness. The laboratory results were as follows: Hct, 25%; Hb, 8 g/dL. The patient was immediately transfused with 1 U of packed red blood cells and 2 U of fresh frozen plasma. A lesion, 20 mm in diameter, consistent with fluid collection, was detected on gray scale sonography (Figure 1), and revision curettage was performed with a Karman cannula after the patient was diagnosed with retained placenta. The patient presented with recurrent bleeding after a week and was referred to our hospital for further investigation and treatment.

**Figure 2:**
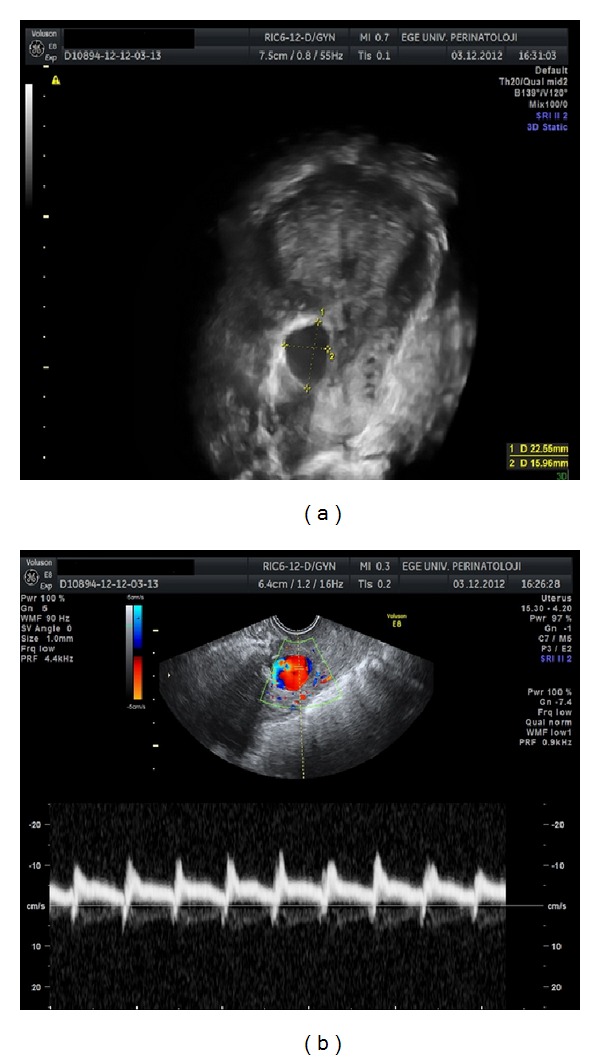
A cystic mass was observed on the right lateral isthmic region with a size of 22 × 16 mm on gray scale and 3D sonography. Color flow and spectral Doppler imaging of the cystic mass revealed marked aliasing and bidirectional flow representing systolic and diastolic blood flow.

**Figure 3:**
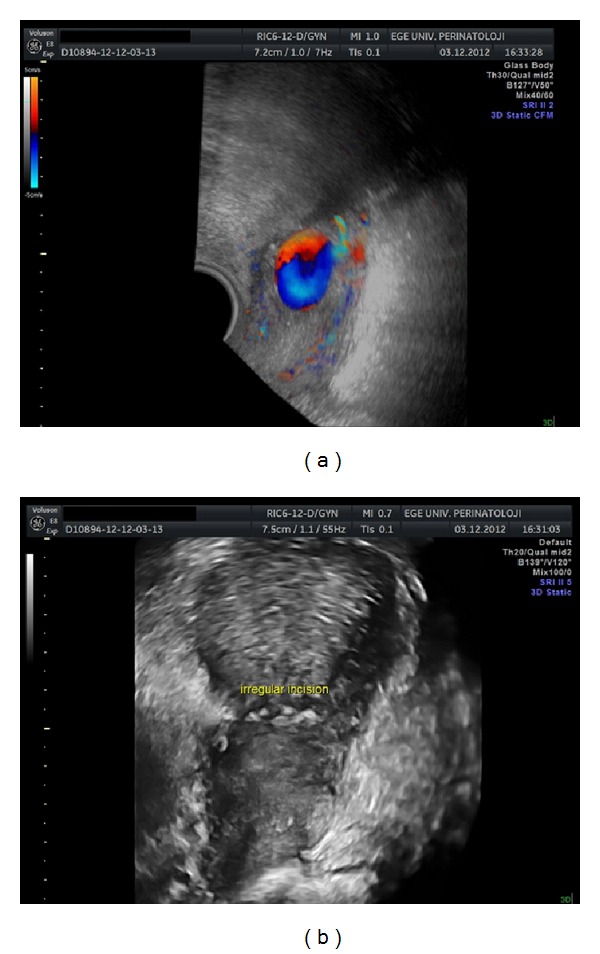
Three-dimensional (3D) sonography or 3D power Doppler mode showed the same cystic mass with a suspicion of irregular incisional track and the highly vascularized cystic mass anastomosed to the uterine vessels.

Systemic evaluation results and vital signs were within normal limits. Gynecological examination revealed normal sized uterus and adnexa without tenderness and vaginal bleeding. Biochemical analyses were unremarkable and hematological findings were as follows: Hct, 30%; Hb, 10 g/dL; WBC, 10000/dL. Gray scale and color Doppler sonography were performed transvaginally and initially showed a normal postpartum uterus and bilateral adnexa. However, careful examination suggested a cystic mass on the right lateral isthmic region with a size of 22 × 16 mm. Color flow and spectral Doppler imaging of the cystic mass revealed marked aliasing and bidirectional flow representing systolic and diastolic blood flow (Figure 2). Three-dimensional (3D) sonography or 3D power Doppler mode showed the same cystic mass with a suspicion of irregular incisional track and the highly vascularized cystic mass anastomosed to the uterine vessels (Figure 3). Pseudoaneurysm was suspected and blood transfusion preparation was initiated for possible emergency surgical intervention. A uterine artery embolization procedure was scheduled the day after the diagnosis. However, on the day of the intervention, the patient experienced excessive vaginal bleeding (approximately 1500 mL) and underwent emergency laparotomy.

**Figure 4:**
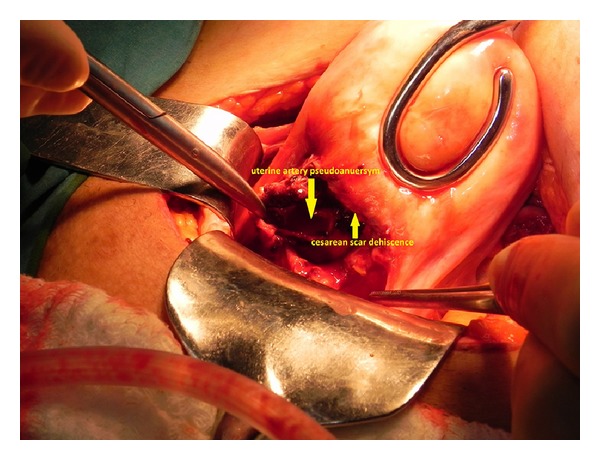
A CSD was inspected and the source of high-flow bleeding was found to be the aneurysmatic formation associated with the right uterine artery, within the uterine cavity.

Abdominal exploration revealed a uterus of normal size and normal adnexa without intra-abdominal bleeding. After the peritoneum of the urinary bladder was detached, the CSD was inspected as both sides of incision were away from each other and the source of high-flow bleeding was found to be the aneurysmatic formation associated with the right uterine artery, within the uterine cavity (Figure 4). The aneurysmatic vessel was resected and retained for pathological evaluation. Subsequently, right uterine artery ligation was performed to preserve fertility. Upon cessation of the bleeding, the lower uterine segment was sutured after the incision was debrided. The patient was transfused with 2 U of packed red blood cells during the perioperative period. On the first postoperative day, hemogram data showed Hct of 23% and Hb of 7.8 g/dL, leading to the transfusion of 2 U of packed red blood cells. Follow-up sonography was unremarkable, and the patient was discharged on the fifth postoperative day.

## 3. Discussion

Postpartum hemorrhage remains one of the major causes of maternal mortality. It occurs in fewer than 5% of all deliveries and accounts for approximately 15% of all maternal deaths [7]. Early or primary postpartum hemorrhage occurs within the first 24 hours postpartum. The primary causes are uterine atony (*∼*70% of cases), retained placental fragments, endometritis, genital laceration, uterine inversion or rupture, and coagulation disorders [8]. Secondary postpartum hemorrhage is defined as excessive bleeding starting any time from 24 hours after delivery up to 6–12 weeks postpartum and most commonly occurs between 8 and 14 days postpartum [3]. Common causes include retained products of conception, subinvolution of the placental bed, and endometritis [9]. Rare causes include pseudoaneurysm of the uterine artery, arteriovenous malformations, CSD, and choriocarcinoma. When the more common causes have been excluded, pelvic angiography may be performed.

UAP should be listed as a possible cause of postpartum hemorrhage after C/S. Trauma to the uterine artery during surgery may cause a defect in the arterial wall, through which arterial blood escapes and diffuses to the adjacent tissues, resulting in the formation of a hematoma. When this hematoma is in continuity with the uterine artery that supplies continuous blood flow, a pseudoaneurysm forms [6]. The absence of a three-layered arterial wall lining in a pseudoaneurysm differentiates it from a true aneurysm. 

In an emergency setting, gray scale ultrasonography is an initial, noninvasive diagnostic tool and may reveal a pseudoaneurysm as a hypoechoic mass associated with the uterine incision. Color and pulsed Doppler ultrasonography may reveal a characteristic to-and-fro pattern, and it has been reported to have a diagnostic sensitivity of 95% [10, 11]. Computed tomography and magnetic resonance imaging can confirm the diagnosis and help rule out other more common causes of delayed postpartum hemorrhage. Angiography remains the standard method for the diagnosis of UAP and may help in the design of definitive treatment strategies [12]. Recently, 3D power Doppler imaging has been used for the diagnosis of UAP. Alboni et al. reported that 3D power Doppler allows the users to define the dimensions of any lesion, detects complex flow patterns, and confirms the relationship between the viscera and the vascular lesion. In our case, we showed that if a specific diagnosis is not suspected, gray scale sonography may lead to misdiagnosis [13]. Color and pulsed Doppler sonography results led to the correct diagnosis of UAP, and 3D sonography and power Doppler imaging revealed the detailed relationship between vascular elements and dehiscence of the cesarean scar. 

A pseudoaneurysm can result in life-threatening profuse postpartum hemorrhage when untreated or treated inadequately. In addition, iatrogenic rupture of the pseudoaneurysm may also occur. Henrich et al. reported a case in which vaginal examination caused rupture of the pseudoaneurysm requiring emergency hysterectomy [14]. Similarly, Eason and Tank reported a case of undiagnosed UAP with abundant bleeding after dilation and curettage that required immediate emergency hysterectomy [2]. Although the present patient underwent similar surgical procedures such as dilatation curettage, only minor bleeding was detected. However, sudden abundant bleeding during the preparation period indicates that UAP should be included as an obstetric emergency. 

Women who have undergone C/S may develop UAP even in the absence of postpartum hemorrhage. Rupture of a pseudoaneurysm can cause severe hemorrhage, although in some cases the rupture is limited by the surrounding tissues, causing intermittent bleeding. In addition, if the pseudoaneurysm is connected to the uterine cavity, postpartum hemorrhage may occur. If the pseudoaneurysm is not connected, hemorrhage may be confined to the abdominal cavity, leading to abdominal pain [6]. 

Extended uterine incision or additional hemostatic suture may be associated with the occurrence of UAP after C/S. Additional sutures often increase the risk of arterial wall damage, resulting in the development of a pseudoaneurysm; however, an extended incision or additional sutures are not always correlated with UAP, and their absence does not preclude the occurrence of this disease [6]. All of these risk factors except repeated C/S were present in our case. 

CSD is estimated to occur in 0.3–1.9% of cases, but bleeding disorders occur only in a small proportion of these cases [15]. Postpartum hemorrhage due to CSD is rarely reported [16]. Baba et al. reported a case of delayed postpartum hemorrhage associated with CSD requiring massive blood transfusion and surgical wound repair [16]. Recently, Sharma and Burbridge reported the results of a study of UAP and CSD. The authors managed UAP with uterine artery embolism; however, conservative treatment for the coexisting CSD caused disseminated intravascular coagulopathy, pelvic abscess, and finally pulmonary embolism [17]. In our case, UAP and CSD were diagnosed concomitantly and the surgical management was adequately given both clinical diagnoses, resulting in the discharge of the patient without complications. 

Uterine artery embolization has become an effective and safe treatment for postpartum hemorrhage, allowing the preservation of reproductive function. Recent reports described the use of thrombin injection directly into the pseudoaneurysm under ultrasound guidance, as a substitute for arterial embolization; however, its indications and effectiveness have not yet been determined [18]. The surgical approach may be more suitable in cases of acute and massive bleeding in which there is no time for embolization and may depend on the specific resources available in each institution. Hysterectomy is one of the surgical options when the preservation of fertility is not important. On the other hand, uterine artery ligation and extirpation of UAP is another surgical choice for preserving fertility.
